# A lost tribe in the city: health status and needs of African asylum seekers and refugees in Hong Kong

**DOI:** 10.1186/s12939-016-0451-4

**Published:** 2016-09-27

**Authors:** William Chi Wai Wong, Sealing Cheng, Eleanor Holroyd, Julie Chen, Kelley Ann Loper, Lynn Tran, Heidi Yin Hai Miu

**Affiliations:** 1 Department of Family Medicine and Primary Care, The University of Hong Kong, Ap Lei Chau Clinic, Rm 3/F., 161 Main Street, Ap Lei Chau, Hong Kong; 2 The Anthropology Department, The Chinese University of Hong Kong, Hong Kong, Hong Kong; 3 School of Clinical Sciences, Auckland University of Technology, Auckland, New Zealand; 4 Bau Institute of Medical and Health Sciences Education, The University of Hong Kong, Hong Kong, Hong Kong; 5 The Department of Law, The University of Hong Kong, Hong Kong, Hong Kong

**Keywords:** Health needs, Access to health, Human rights, Refugees, Hong Kong

## Abstract

**Background:**

Hong Kong’s resistance to be a signatory of the 1951 Geneva Convention and lack of domestic policies in this area has resulted in restrictions on access to healthcare amongst asylum seekers and refugees (ASRs). Using social determinants of health framework this study sought to identify health practices, problems and needs of African ASRs in Hong Kong.

**Methods:**

A cross-sectional survey comprising of six domains including health status, health-seeking behaviour and social experience targeted at adult African ASRs in Hong Kong was conducted through three local non-governmental organisations between February and April 2013. Outpatient care and inpatient care in the past 12 months were used as proxy measures of general and severe ill health respectively. Associations between the determinants of health factors with general or severe health was explored through logistic regressions.

**Results:**

Majority of 374 participants were young, single, educated males having been in Hong Kong for over 5 years. A third of ARS (36.1 %) screened positive for depression. Most reported problems related to basic necessities (64.7–78.6 %) and access to health services (72.2 %). ASRs with relatively less education, health awareness or higher risk behaviours were less likely to have obtained outpatient or inpatient services. African ASRs reporting problems with case officers (aOR = 2.80; 95 % CI = 1.35-5.79) or illness in the past 30 days (aOR = 6.00; 95 % CI = 2.94-12.25) were more likely to report general ill health. Similarly, problems with the case officers (aOR = 3.76; 95 % CI = 1.97-7.18) and self-reported illness in the past 30 days (a​OR = 3.32; 95 % CI= 1.68-6.57) were also significantly associated with severe ill health. At the health system level, those who reported experiencing difficulties accessing the medical services in Hong Kong are 3.29 (95 % CI = 1.48-7.31) and 4.12 (95 % CI = 1.73-9.79) times as likely to report general and severe ill health respectively.

**Conclusion:**

The host government should have moral and ethical obligations to attend to the health needs of ASRs. Evidently a number of structural and health system factors have significantly impacted the health of African ASRs in Hong Kong. Changes to current policies regarding how African ASRs are handled whilst in Hong Kong but, more immediately, improvements in healthcare access are needed.

## Background

Every year thousands of refugees across the world are displaced because of war, violence, or persecution. The United Nations High Commissioner for Refugees (UNHCR) estimates that there are approximately 59.5 million forcibly displaced persons worldwide [1]. In Hong Kong the number of asylum seekers and refugees (ASRs) including torture claimants has increased from 6699 cases in 2014 to 10,922 in 2015 [2]. Approximately 9 % of these ASRs are from Africa [3].

Historically, Hong Kong has resisted being a signatory to the *1951 Convention relating to the Status of Refugees*, an international legal instrument establishing rights and protection for refugees resulting in the prohibition of Hong Kong refugees from working or studying [4]. However, has been a co-signatory to the UN’s Convention against Torture since 1992, which prohibits refoulement of individuals when there is possibility that the individual would be subjected to torture or cruelty [3, 4]. Despite the Unified Screening Mechanism (USM) put in place to screen refugee protection claims, the recognition rate for refugee protection has been close to zero [5]. In essence, refugees are trapped in Hong Kong unable to get their claims processed and also without any legal means to financially support themselves.

The political, social, and economic constraints imposed on ASRs in Hong Kong exert profound effects to their health and wellbeing. In 2015, a reported 232 asylum seekers were arrested for working illegally, while 1113 were detained for other criminal offences [2]. This is not surprising as ASR remain in limbo with the Hong Kong government providing only basic necessities. Housing is subsidized through financial assistance of HK$1200 (US$154.51) per month and is paid directly to landlords [6]. Food is provided as food bags which contain only HK$40 (USD$5.88) worth of food meant to last for 5 to 10 days [7]. For health access, ASRs are liable for exemption from usual expenses of routine medical care in the public health sector by presenting a Medical Waiver issued from the Social Work Department; however these are considered on a case-by-case basis [8].

Displacement is often a considerable traumatising disruption to the lives of those involved, and the can inflict a wide range of health problems which are not dissimilar to general populations. Health related problems such as injuries, psychosocial problems, illnesses related to poor sanitation and nutrition can be associated with displacement [9–12]. Management of non-communicable diseases and growth and development of younger populations are also notable problems [9, 10]. Further compounding this is the cultural and language barriers that arise when ASRs try to access the healthcare system. This has been assessed with regards to the interaction of the ASRs with the healthcare systems; incorporating socio-cultural dimensions and health seeking behaviours of the ASR populations, as well as capacity of the healthcare systems to facilitate for ASR populations [13–15].

The objectives of the study were to identify the health practices, problems, and needs of African ASRs in Hong Kong, specifically exploring the influence of socio-cultural, political and economic conditions had on the lives of African ASRs in accordance to health status and health needs.

## Methods

The study was comprised of two parts: an initial interview-based qualitative study to explore the nature of life as an African refugee in Hong Kong; followed by a cross sectional quantitative survey. The findings from the qualitative study formed the basis for the subsequent survey. Only the results of the quantitative component will be reported in this paper. Researchers worked in collaboration with three local organisations: The Vine Church, Vision First, and the African Community Centre for recruitment and data collection between February and April 2013. The three local organisations have a longstanding history working with ASRs in Hong Kong; providing support, legal advice, education, and other services. Potential participants were invited by the collaborating organisations through outreach or while they were attending their regular service. They were invited to complete the survey if they were ≥18 years of age and able to read and write in English, French, or Somali. Participants provided verbal consent and received HK$50 (US$1 = HK7.76) remuneration for their participation.

### Conceptual framework

**Fig. 1:**
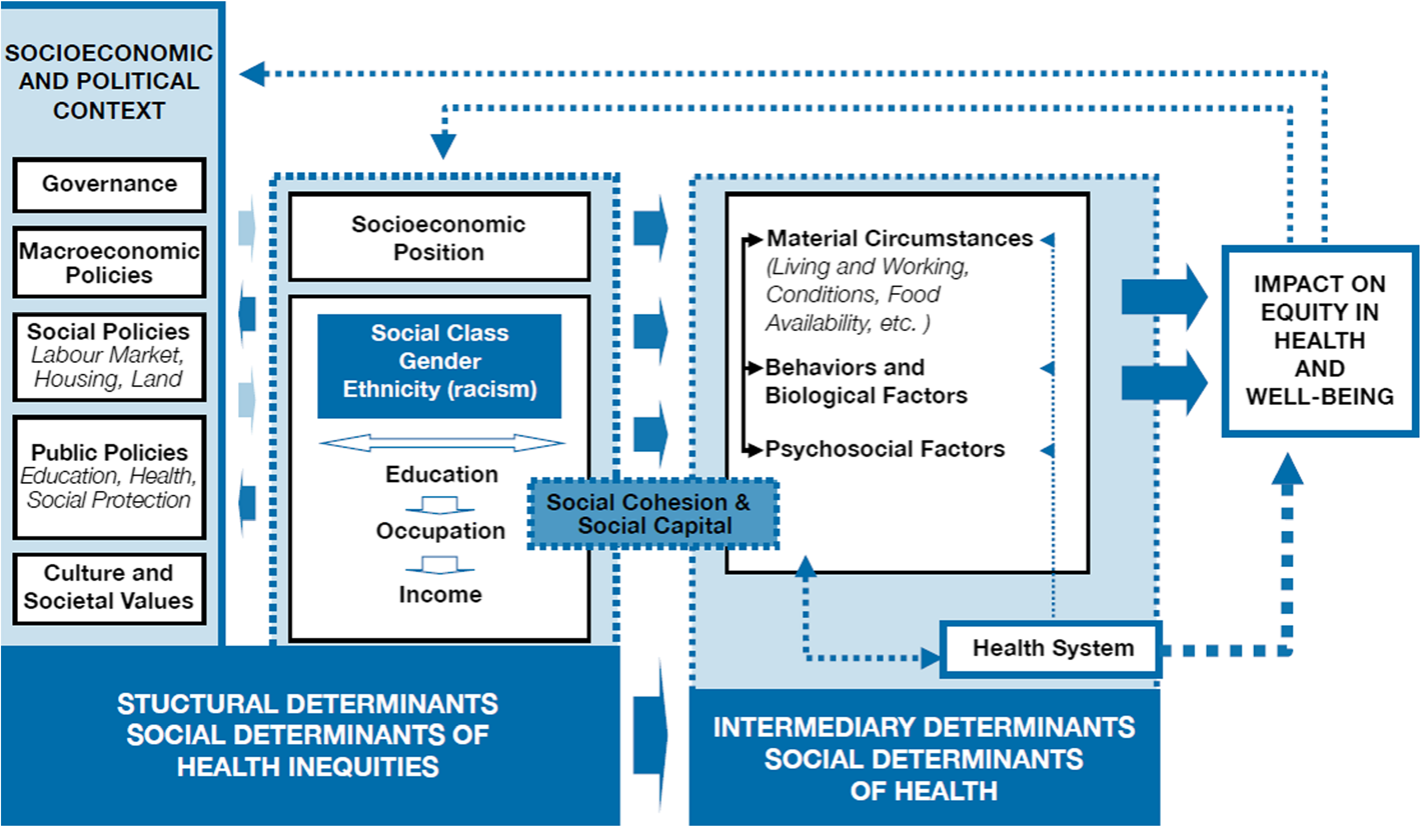
Conceptual framework adapted from the WHO [16] to depict how social determinants of health can interact to impact health and wellbeing of African ASRs in Hong Kong

The Conceptual Framework for the Social Determinants of Health (CSDH) Framework (Fig. 1.) developed by the World Health Organization (WHO) [16] was used to provide an inclusive, overarching guide to explore factors that impact health and wellbeing of African ASRs in Hong Kong. The CSDH maps determinants of health to health outcomes (Fig. 1. Adopted from [16]) describing the socio-political context as the mechanism that produces and perpetuates social hierarchies, including labour market forces, education systems, political institutions, and cultural/societal values (Fig. 1.). Social hierarchies shape the socioeconomic position of an individual and are the systemic causes behind inequities in health in turn influencing the distribution of downstream/intermediary determinants consisting of (a) Behavioural and biological factors; (b) Material Circumstances; (c) Psychosocial Circumstances; and (d) the Health system factors.

### Survey instrument

The survey instrument comprised 55 questions seeking information across six domains (a) demographics, (b) health status, (c) health-seeking behaviour, (d) social experiences, (e) access to food and lifestyle, and (f) sexual and reproductive health. To capture social experiences the Everyday Discrimination Scale (EDS), a validated tool consisting of 5 questions which assessed the frequency of encounters and responses to daily discrimination was used (Table 1.) [17]. The PHQ2 was also included, and is a validated screening tool of 2 questions used to screen for symptoms of depression [18]. Originally drafted in English, the survey instrument was pilot-tested for face validity with eight African ASRs independent of the main study; minor revisions were then made accordingly. The surveys were then translated to both French and Somali, proofread, and edited by two bilingual native speakers of French; and back-translated by Somali-speaking African ASR volunteers respectively. To explore the health status of African refugees, respondents were asked about (a) outpatient services, such as treatment and medications within the past 12 months; and (b) inpatient services, i.e. hospital admission within the past 12 months. These were used as proxy measures of general and severe ill health respectively.

**Table 1:** Basic descriptive of African ASRs in Hong Kong, assessing different demographic factors as well as the social determinants of health as defined by the CSDH framework (Solar & Irwin, 2010)

Socio-demographics characteristics	
Gender	
Male	292 (78.0 %)
Female	82 (21.9 %)
Age Group (Mean: 31.52, SD: ±7.41)	
18–27 yrs	107 (28.6 %)
28–37 yrs	194 (51.9 %)
38–47 yrs	66 (17.6 %)
48 yrs +	7 (1.9 %)
Received inpatient services^a^	
Yes	146 (39.0 %)
No	228 (61.0 %)
Place of origin	
Northern & Eastern Africa	144 (38.5 %)
Central and Southern Africa	79 (21.1 %)
Western Africa	151 (40.4 %)
Length of Residence in Hong Kong	
Less than 5 years	280 (74.9 %)
5 to 10 years	89 (23.8 %)
11 years plus	5 (1.3 %)
Received outpatient services^a^	
Yes	217 (58.0 %)
No	157 (42.0 %)
Intermediary factors	
Material/Living Circumstances	
Problems with Accommodation	
No	80 (21.4 %)
Yes	294 (78.6 %)
Problems with food packages	
No	132 (35.3 %)
Yes	242 (64.7 %)
Problems with case officer	
No	174 (46.5 %)
Yes	200 (53.5 %)
Biological/Behavioural Circumstances	
Alcohol consumption^bc^	
None	268 (71.7 %)
Infrequently	57 (15.2 %)
Frequently	49 (13.1 %)
Smoking^bd^	
None	260 (69.5 %)
Light Smoker	24 (6.4 %)
Heavy Smoker	90 (24.1 %)
Recreational Drugs^b^	
No	342 (91.4 %)
Yes	32 (8.6 %)
Exercise^be^	
Little or none	276 (73.8 %)
Frequent	98 (26.2 %)
Multiple Sex partners^bf^	
None	201 (53.7 %)
Only one	117 (31.3 %)
More than one	56 (15.0 %)
Illness, Injury, or symptoms^bf^	
No	193 (51.6 %)
Yes	181 (48.4 %)
Chronic illness^bf^	
No	223 (59.6 %)
Yes	151 (40.4 %)
Psychosocial Factors	
Religion	
Atheist	14 (3.7 %)
Christian/Catholic	211 (56.4 %)
Muslim	142 (38.0 %)
Other	7 (1.9 %)
Marital Status	
Single	252 (67.4 %)
Married	71 (19.0 %)
Divorced/Separated/Widowed	51 (13.6 %)
Living Companion	
Alone	286 (76.5 %)
With Family	65 (17.4 %)
With Others	23 (6.1 %)
General health^f^	
Very Good/Excellent	72 (19.2 %)
Good	114 (30.5 %)
Fair to Poor	188 (50.3 %)
Health vs. locals of same age^f^	
Better or Much Better	162 (43.3 %)
Same	133 (35.6 %)
Worse or Much Worse	79 (21.1 %)
Health vs. prior arrival in HK^f^	
Better or Much Better	148 (39.6 %)
Same	110 (29.4 %)
Worse or Much Worse	116 (31.0 %)
PHQ2 Score (Depression screen)	
Below 2	239 (63.9 %)
Equal or above 2	135 (36.1 %)
Healthsystem factors	
Satisfaction with Medical Services	
Not used	87 (23.3 %)
Fair/Poor	105 (28.1 %)
Good-Excellent	182 (48.6 %)
Difficulties Accessing Medical Facilities	
No	104 (27.8 %)
Yes	270 (72.2 %)
Structural factors	
Socio-economic Position	
ASR Status	
Refugee	32 (8.6 %)
Asylum Seeker Claimant	172 (46.0 %)
Torture Claimant	115 (30.7 %)
Asylum Seeker & Torture Claimant	55 (14.7 %)
Education Level in country of origin	
None	55 (14.7 %)
Primary	87 (23.3 %)
High School	177 (47.3 %)
University or above	55 (14.7 %)
Occupation in country of origin	
Professional	124 (33.1 %)
Supervisory	57 (15.2 %)
Skilled manual worker	74 (19.8 %)
Semi/Unskilled manual worker	44 (11.8 %)
Casual worker or unemployed	75 (20.1 %)
Discrimination	
Everyday Discrimination Scale: Mean = 12.87; SD = ±7.01	

^a^Reported in the past 12 months; ^b^Reported in the past 30 days; ^c^Alcohol Consumption – None = does not drink, Infrequently = <5 drinks/week, Frequently= > 5 drinks/week; ^d^Smoking – Light Smokers = <10 cigarettes per day, Heavy Smokers= > 10 Cigarettes per day; ^e^Exercise – Little or none = <15 days with exercise, Frequent= > 15 days with exercise; ^f^Based on the participants own judgement

### Data analysis

Survey questions were mapped to the CSDH framework and analysed with descriptive statistics. Associations between the determinants of health factors with severe or general health were explored through univariate and multivariable logistic regression. The adjusted odds ratios were firstly adjusted by gender to account for the differences between male and female participants. Data were analysed using SPSS (Version 23.0). All basic assumptions were tested and met.

## Results

### Demographics and social experiences of African ASRs in Hong Kong

Participant demographics and background characteristics can be found in Table 2. The majority of the 374 participants were male (78.1 %), single (67.4 %), and between 28 and 37 years (81.5 %) (Table 2). Prior to arrival in Hong Kong, 48.4 % had professional and supervisory roles, with 62 % also having obtained high school education or above. Most participants reported problems with their food bags (64.7 %), accommodation (78.6 %), and half (53.5 %) cited problems with the case officer who manages their ASR application. Of those that had accessed healthcare facilities, 48.7 % reported the services to be “Good” to “Excellent”, though 72.2 %, reported having difficulties accessing medical facilities, particularly with obtaining the medical waiver.

**Table 2:** Unadjusted (OR) and Adjusted (aOR) Odds Ratios of factors significantly associated with general ill health (Receiving outpatient services in past 12 months) and severe ill health (obtaining inpatient services within past 12 months) in African ASRs in HK respectively

	Outpatient services		Inpatient services	
	OR (95 % C.I.)	aOR (95 % C.I.)	OR (95 % C.I.)	aOR (95 % C.I.)
Gender	0.8 (0.49–1.30)	0.55 (0.24–1.28)	0.71 (0.43–1.20)	0.39 (0.17–0.88)
Intermediary Determinants				
Material Circumstances				
Housing/accommodation Problems	1.96 (1.19–3.23)*	0.61 (0.25–1.49)	–	–
Problems with case officer	1.94 (1.28–2.95)*	2.80 (1.35–5.79)*	2.08 (1.36–3.19)*	3.76 (1.97–7.18)*
Individual and Behavioural circumstances				
Age	–	–	0.97 (0.94–0.99)*	0.94 (0.89–0.98)*
Place of Origin (North & Eastern Africa)				
Central and Southern Africa	1.16 (0.67–2.01)	1.19 (0.51–2.82)	0.57 (0.32–1.04)	0.53 (0.22–1.23)
Western Africa	1.7 (1.07–2.72)*	1.35 (0.62–2.97)	1.15 (0.72–1.83)	0.89 (0.44–1.81)
Alcohol consumption^ab^ (None)				
Infrequently	–	–	0.59 (0.32–1.10)	0.38 (0.16–0.90)*
Frequently	–	–	0.74 (0.39–1.40)	0.94 (0.37–2.42)
Smoking^ac^ (None)				
Light Smoker	1.25 (0.52–3.03)	1.27 (0.34–4.73)	–	–
Heavy Smoker	0.52 (0.32–0.85)*	0.52 (0.22–1.28)	–	–
Recreational Drugs^a^	2.79 (1.17–6.63)*	3.48 (0.92–13.17)	–	–
Frequent Exercise^ad^	–	–	2.07 (1.30–3.30)*	1.98 (1.01–3.90)*
Multiple Sex partners^a^ (None)				
One	0.7 (0.44–1.12)	0.48 (0.23–0.97)*	0.38 (0.23–0.62)*	0.24 (0.11–0.48)*
More than one	0.5 (0.28–0.92)*	0.49 (0.18–1.31)	0.61 (0.33–1.12)	0.59 (0.23–1.49)
Illness, Injury, or symptoms^ae^	9.1 (5.59–14.81)*	6.00 (2.94–12.25)*	4.15 (2.66–6.47)*	3.32 (1.68–6.57)*
Chronic illness^ae^	9 (5.32–15.22)*	3.47 (1.66–7.27)*	3.78 (2.44–5.86)*	2.18 (1.10–4.30)*
Psychosocial Factors				
Marital Status (Single)				
Married	1.84 (1.05–3.25)*	1.45 (0.50–4.22)	1.67 (0.98–2.84)	2.60 (1.00–6.75)*
Separated	0.74 (0.41–1.36)	2.98 (1.02–8.68)*	0.61 (0.32–1.20)	2.20 (0.78–6.24)
Living Companion (Alone)				
With Family	1.38 (0.79–2.41)	0.97 (0.34–2.75)	1.5 (0.87–2.58)	0.90 (0.36–2.25)
Others	2.92 (1.05–8.07)*	2.03 (0.41–10.01)	1.91 (0.81–4.48)	0.92 (0.27–3.15)
Perception of general health (Fair/Poor)				
Very Good/Excellent	–	–	1.79 (1.03–3.11)*	1.81 (0.80–4.10)
Good	–	–	1.28 (0.79–2.07)	1.36 (0.66–2.80)
PHQ2 Score (Depression screen)	2.4 (1.53–3.77)*	1.08 (0.89–1.31)	1.17 (0.76–1.81)	0.96 (0.81–1.14)
Health System Factors				
Difficulties accessing medical facilities (Yes)	9.69 (5.64–16.64)*	3.29 (1.48–7.31)*	7.55 (3.95–14.43)*	4.12 (1.73–9.79)*
Satisfaction with Medical Services (Not used)				
Fair/Poor	7.90 (4.06–xxx)	4.66 (1.65–13.13)*	11.29 (4.96–25.68)*	8.83 (2.83–27.53)*
Good-Excellent	10.58 (5.69–19.68)	5.73 (2.01–16.35)*	8.1 (3.70–17.73)*	4.08 (1.35–12.32)*
Structural Determinants				
Socioeconomic Position				
Status in HK (refugee)				
Asylum Seeker	0.63 (0.27–1.44)	1.65 (0.56–4.87)	0.94 (0.44–2.00)	2.53 (0.92–6.95)
Torture Claimant	0.53 (0.22–1.24)	0.43 (0.14–1.33)	0.73 (0.33–1.60)	1.79 (0.62–5.21)
Asylum seeker & Torture Claimant	0.26 (0.10–0.67)*	1.71 (0.41–7.11)	0.19 (0.07–0.54)*	0.49 (0.13–1.88)
Education level in country of origin (None)				
Primary	0.15 (0.07–0.35)*	0.73 (0.19–2.80)	2.78 (1.29–5.99)*	0.79 (0.22–2.87)
Secondary	0.44 (0.21–0.91)*	1.03 (0.27–3.90)	2.24 (1.10–4.54)*	0.64 (0.18–2.23)
University or above	0.68 (0.35–1.32)	0.72 (0.15–3.49)	3.72 (1.62–8.52)*	0.71 (0.17–3.00)
Occupation in country of origin (Professional)				
Supervisory/Junior Managerial	1.73 (0.88–3.42)	2.90 (1.14–7.36)*	0.85 (0.45–1.60)	1.29 (0.54–3.07)
Skilled Manual Worker	0.51 (0.29–0.92)*	1.11 (0.41–2.98)	0.46 (0.25–0.87)*	0.86 (0.33–2.20)
Semi/Un-skilled Manual Worker	0.68 (0.34–1.35)	2.09 (0.71–6.17)	0.72 (0.35–1.46)	2.26 (0.79–6.49)
Casual/Unemployed	1.2 (0.66–2.17)	2.76 (1.15–6.62)*	0.93 (0.52–1.67)	1.99 (0.87–4.56)
Discrimination				
Everyday Discrimination Scale	1.04 (1.01–1.08)*	1.00 (0.96–1.05)	–	–

^a^Reported in the past 30 days; ^b^Alcohol Consumption – None = does not drink, Infrequently = <5 drinks/week, Frequently= > 5 drinks/week; ^c^Smoking – Light Smokers = <10 cigarettes per day, Heavy Smokers= > 10 Cigarettes per day; ^d^Exercise – No exercise and infrequent exercise was combined to give two groups, Frequent exercise = > 15 days with exercise; ^e^Based on the participants own judgement; * *p* < 0.05

### Health needs and health behaviours of African ASRs

A majority of participants reported positive health behaviours (Table 2.) including being non-smokers (69.5 %), non-drinkers (71.7 %), and non-users of recreational drugs (91.4 %), though 73.8 % undertook little or no exercise and 15.0 % report having more than one sex partner. About 58.0 % of participants had obtained outpatient services as a result of symptoms, illness, or injury in the last 12 months (reflecting their general ill health), and 39.0 % had received inpatient services in the last 12 months (reflecting severe ill health). Half of participants (50.3 %) rated their health as “fair” or “poor”. Half of respondents reported to have had illness, symptoms of injury in the last 30 days (48.4 %) or reported chronic illness (40.4 %).

### Factors Associated with General and Severe Ill health

The association of different variables in relation to severe and general ill health, after adjusting for gender, can be found in Table 3.

**Table 3:** Unadjusted (OR) and Adjusted (aOR) Odds Ratios of factors significantly associated with general ill health (Receiving outpatient services in past 12 months) and severe ill health (obtaining inpatient services within past 12 months) in African ASRs in HK respectively (n=374)

	Outpatient services		Inpatient services	
	OR (95 % C.I.)	aOR (95 % C.I.)	OR (95 % C.I.)	aOR (95 % C.I.)
Gender	0.8 (0.49–1.30)	0.55 (0.24–1.28)	0.71 (0.43–1.20)	0.39 (0.17–0.88)
Intermediary Determinants				
Material Circumstances				
Housing/accommodation Problems	1.96 (1.19–3.23)*	0.61 (0.25–1.49)	-	-
Problems with case officer	1.94 (1.28–2.95)*	2.80 (1.35–5.79)*	2.08 (1.36–3.19)*	3.76 (1.97–7.18)*
Individual and Behavioural circumstances				
Age	-	-	0.97 (0.94–0.99)*	0.94 (0.89–0.98)*
Place of Origin (North & Eastern Africa)				
Central and Southern Africa	1.16 (0.67–2.01)	1.19 (0.51–2.82)	0.57 (0.32–1.04)	0.53 (0.22–1.23)
Western Africa	1.7 (1.07–2.72)*	1.35 (0.62–2.97)	1.15 (0.72–1.83)	0.89 (0.44–1.81)
Alcohol consumption^ab^ (None)				
Infrequently	-	-	0.59 (0.32–1.10)	0.38 (0.16–0.90)*
Frequently	-	-	0.74 (0.39–1.40)	0.94 (0.37–2.42)
Smoking^ac^ (None)				
Light Smoker	1.25 (0.52–3.03)	1.27 (0.34–4.73)	-	-
Heavy Smoker	0.52 (0.32–0.85)*	0.52 (0.22–1.28)	-	-
Recreational Drugs^a^	2.79 (1.17–6.63)*	3.48 (0.92–13.17)	-	-
Frequent Exercise^ad^	-	-	2.07 (1.30–3.30)*	1.98 (1.01–3.90)*
Multiple Sex partners^a^ (None)				
One	0.7 (0.44–1.12)	0.48 (0.23–0.97)*	0.38 (0.23–0.62)*	0.24 (0.11–0.48)*
More than one	0.5 (0.28–0.92)*	0.49 (0.18–1.31)	0.61 (0.33–1.12)	0.59 (0.23–1.49)
Illness, Injury, or symptoms^ae^	9.1 (5.59–14.81)*	6.00 (2.94–12.25)*	4.15 (2.66–6.47)*	3.32 (1.68–6.57)*
Chronic illness^ae^	9 (5.32–15.22)*	3.47 (1.66–7.27)*	3.78 (2.44–5.86)*	2.18 (1.10–4.30)*
Psychosocial Factors				
Marital Status (Single)				
Married	1.84 (1.05–3.25)*	1.45 (0.50–4.22)	1.67 (0.98–2.84)	2.60 (1.00–6.75)*
Separated	0.74 (0.41–1.36)	2.98 (1.02–8.68)*	0.61 (0.32–1.20)	2.20 (0.78–6.24)
Living Companion (Alone)				
With Family	1.38 (0.79–2.41)	0.97 (0.34–2.75)	1.5 (0.87–2.58)	0.90 (0.36–2.25)
Others	2.92 (1.05–8.07)*	2.03 (0.41–10.01)	1.91 (0.81–4.48)	0.92 (0.27–3.15)
PHQ2 Score (Depression screen)	2.4 (1.53–3.77)*	1.08 (0.89–1.31)	1.17 (0.76–1.81)	0.96 (0.81–1.14)
Health System Factors				
Difficulties accessing medical facilities (Yes)	9.69 (5.64–16.64)*	3.29 (1.48–7.31)*	7.55 (3.95–14.43)*	4.12 (1.73–9.79)*
Satisfaction with Medical Services (Not used)				
Fair/Poor	7.90 (4.06-xxx)	4.66 (1.65–13.13)*	11.29 (4.96–25.68)*	8.83 (2.83–27.53)*
Good-Excellent	10.58 (5.69–19.68)	5.73 (2.01–16.35)*	8.1 (3.70–17.73)*	4.08 (1.35–12.32)*
Structural Determinants				
Socioeconomic Position				
Status in HK (refugee)				
Asylum Seeker	0.63 (0.27–1.44)	1.65 (0.56–4.87)	0.94 (0.44–2.00)	2.53 (0.92–6.95)
Torture Claimant	0.53 (0.22–1.24)	0.43 (0.14–1.33)	0.73 (0.33–1.60)	1.79 (0.62–5.21)
Asylum seeker & Torture Claimant	0.26 (0.10–0.67)*	1.71 (0.41–7.11)	0.19 (0.07–0.54)*	0.49 (0.13–1.88)
Education level in country of origin (None)				
Primary	0.15 (0.07–0.35)*	0.73 (0.19–2.80)	2.78 (1.29–5.99)*	0.79 (0.22–2.87)
Secondary	0.44 (0.21–0.91)*	1.03 (0.27–3.90)	2.24 (1.10–4.54)*	0.64 (0.18–2.23)
University or above	0.68 (0.35–1.32)	0.72 (0.15–3.49)	3.72 (1.62–8.52)*	0.71 (0.17–3.00)
Occupation in country of origin (Professional)				
Supervisory/Junior Managerial	1.73 (0.88–3.42)	2.90 (1.14–7.36)*	0.85 (0.45–1.60)	1.29 (0.54–3.07)
Skilled Manual Worker	0.51 (0.29–0.92)*	1.11 (0.41–2.98)	0.46 (0.25–0.87)	0.86 (0.33–2.20)
Semi/Un-skilled Manual Worker	0.68 (0.34–1.35)	2.09 (0.71–6.17)	0.72 (0.35–1.46)	2.26 (0.79–6.49)
Casual/Unemployed	1.2 (0.66–2.17)	2.76 (1.15–6.62)*	0.93 (0.52–1.67)	1.99 (0.87–4.56)
Discrimination				
Everyday Discrimination Scale	1.04 (1.01–1.08)*	1.00 (0.96–1.05)	-	-

(*) – p < 0.05

(^a^) - Reported in the past 30 days; (^b^) - Alcohol Consumption – None = does not drink, Infrequently = <5 drinks/week, Frequently= > 5 drinks/week; (^c^) - Smoking – Light Smokers = <10 cigarettes per day, Heavy Smokers= > 10 Cigarettes per day; (^d^) - Exercise – No exercise and infrequent exercise was combined to give two groups, Frequent exercise = > 15 days with exercise; (^e^) - Based on the participants own judgement

#### Intermediary level factors

In relation to material circumstances, reporting problems with the case officer was found to be significantly associated with general and severe ill health. Those reporting problems with case officers were 2.80 times (95 % confidence Interval (95 % CI) = 1.35–5.79) as likely to have experienced general ill health that those without. They were also 3.76 times as likely to report severe ill health (95 % CI = 1.97–7.18). Age showed a small albeit significant association to severe illness (Adjusted Odds Ratio (aOR) = 0.94; 95 % CI = 0.89–0.98). Those who consumed alcohol = <5 drinks/week had reduced odds of severe health than non-drinkers (aOR = 0.38; 95 % CI = 0.16–0.90), whereas those who took part in frequent exercise also displayed significant odds (aOR = 1.98; 95 % CI = 1.01–3.90). aOR of general and severe ill health for those with one sex partner was 0.48 (95 % CI = 0.23–0.97) and 0.24 (95 % CI = 0.11–0.48) compared to those with no sex partners respectively. Those who reported illness in the last 30 days also showed an association to general ill health (aOR = 6.00; 95 % CI = 2.94–12.25) and severe ill health (aOR = 3.32; 95 % CI = 1.68–6.57). Those reporting chronic disease revealed significant associations to general (aOR = 3.47; 95 % CI = 1.66–7.27) and severe (aOR = 2.18; 95 % CI = 1.10–4.30) ill health compared to no chronic illness. Further, those reporting self-perceived health to be “very good” to “excellent” were 2.30 times as likely (95 % CI = 1.10–4.81) as those who perceived their own health to be “poor” to report severe ill health. This is also the case for those that perceived their health to be “good” (OR = 1.96, 95 % CI = 1.03–3.74). PHQ2 was not a significantly associated. Further, those reporting self-perceived health to be “Very good-to-excellent” were 1.81 times as likely (95 % CI = 0.80-4.10) as those who perceived their own health to be “Fair-to-poor” to report severe ill health. This relationship was also the case for those that perceived their health to be “Good” (OR = 1.36, 95 % CI = 0.66-2.80).

#### Health system level and structural factors

Those who experience difficulties accessing medical facilities showed association to general (aOR = 3.29; 95 % CI = 1.48–7.31) and severe (aOR = 4.12; 95 % CI = 1.73–9.79) ill health compared to those who did not. At the structural level those reporting casual roles or unemployment prior to arrival in Hong Kong were 2.53 times more predisposed to general ill health as those with previous professional occupations (95 %CI = 1.09–5.85). This was also found for those reporting supervisory or junior managerial occupations (aOR = 2.90; 95 % CI = 1.14-7.36). The EDS not significantly associated with general ill health upon controlling for other variables.

## Discussion

The WHO CSDH framework is a useful tool to understand how the migration processes and host society’s legislative policies impact the salient individual, inter-professional, interpersonal and socio political factors that impinge on the health status, needs and behaviours of African ASRs in Hong Kong.

### Intermediary factors

Intermediary factors significantly related to ill health of African ASRs in Hong Kong include age, drug use, exercise, sexual health behaviours, previous illness, and prevalence of chronic illness. How these factors are directly related to health have been examined in the literature [19–21]. The result of this is increased risk of general ill health and chronic illness [10, 20]. In addition to this, substance abuse, including alcohol is a notable problem in ASRs as it is thought to be related to posttraumatic stress, prolonged instability and hardship due to displacement. However, further in-depth investigations should be made to assess the relationship of specific health behaviours with ill health.

Self-perceived health is able to incorporate a more holistic definition of health compared to medical records and diagnosis alone; such as contextual and psychosocial factors and has been studied amidst refugee and immigrant populations [19, 22]. In the univariate analysis, it was revealed that participants reporting “Very good-to-Excellent” health are associated with receiving inpatient services in the last 12 months. It is possible that those who have received medical services had means to access adequate treatment; hence perceived their health to be better compared to those who have not been able to access healthcare. In addition, they may also have access to greater resources to engage other biological, social or psychological support, all of which can all be encompassed within self-perceived health [23]. Although, this was no longer statistically significant when other variables were included, the unusual association is maintained. We contend that further investigations should assess this in more depth.

Unique to the Hong Kong context was experiencing of problems with the case officer managing the ASR claim. Case officers can be considered as the interface between the ASR populations and the refugee screening system. However the rigid policies, combined with a general hostility towards ASRs creates a drawn-out process for ASRs for whom there is a little hope of being recognised [3]. ASRs are rendered financially crippled, socially and culturally devoid of identity, and suffering negative health consequences. The Hong Kong Government claims that strict policies prevent exploitation and abuse of the economy; [24] it has been shown that ASRs do not compete with local residents. Rather, they create niche markets which arguably help to keep small local businesses afloat in an increasingly competitive economic environment [24].

### Healthcare system and structural factors

Analysis of health system factors shows an association between difficulties accessing medical services to general and severe health. Without being able to establish causal relationship, further analysis will be required. However, ASRs who have accessed medical services for recent bouts of ill health would be most able to reflect on the difficulties experienced with the medical services. Obtaining of a medical waiver to waive their healthcare costs is also a unique situation to Hong Kong. Unfortunately, medical waivers are only permitted on a case-by-case basis. Without it, ASRs need to pay out-of-pocket with the medical fees adding to the financial strain which can prolong or lead to delayed treatment [25, 26].

Structural factors associated with poor health outcomes include occupation prior to arrival in Hong Kong. Downward occupational shift is a well-established phenomenon for displaced populations, [27] particularly due to complete prohibition of employment. Here, ASRs are unable to escape the financially dependent status regardless of previous skills, experience, and expertise. Many may be forced into hazardous, unskilled intensive forms of informal labour to make ends meet even at the risk of arrest and imprisonment [24]. This can also indirectly compound already negative attitudes towards ASRs, such as that already seen in China [28]. Among ASR who were previously professionals or in managerial positions, such a decline in socioeconomic position invariably affects physical and mental health due to the nature of their work, but also psychological strain. The risk of mental distress on African ASRs in Hong Kong will be reported elsewhere.

The importance of this current situation is manifold. At the local context, it is arguable that there has been a breach in the integrity of the healthcare system which prides itself on a care-based approach and has retained the principle to promote health of the community since 1974 [29]. However, the current lack of healthcare access for African ASR populations represents a breach of this principle, as a result of the constitutional barriers that prevent healthcare access. At an epidemiological level, the poor health status of ASRs in Hong Kong is significant public health matter due to propensity for infectious disease spread, particularly in the sense city of Hong Kong [21, 30]. From a humanitarian perspective, the institutional environment in which African ASRs live in Hong Kong breaches their rights to health, especially as Hong Kong has signed and ratified the International Covenant on Economic, Social and Cultural Rights (ICESCR) [31]. The United Nations itself has raised concerns regarding the rights of ASRs in Hong Kong, including the right to health; expressing concern to *“prevalent and widespread discrimination against some disadvantaged and marginalized groups”* including ASRs [32]. Hence, there is an urgent need to reconsider the perceived roles of ASRs in Hong Kong’s economy and society and to reassess the deficiencies of the current system with respect to economic and social constraints.

### Implications

Our intermediary factors suggest that Hong Kong urgently needs to re-assess its obligation to facilitate access to healthcare for this vulnerable population,  due to the barriers to service provision imposed by the medical waiver, as well as relationships with the case worker. Though we argue for more in-depth analysis into the barriers in access medical services for ASRs in Hong Kong, there is a need to make the mechanisms of medical access more transparent and enabling for ASR populations as well as transferring this to current health service delivery personnel.

In light of the identified structural factors that directly and indirectly impact the health and wellbeing of ASRs in Hong Kong, there is also a need to reassess the screening mechanism where fairness, transparency, and efficiency are currently inadequate. There needs to be a major shift in the conceptualization of the position of ASRs in Hong Kong which should be translated into, and operationalized, as government policy. At the most fundamental level, Hong Kong needs to not only fulfil its commitment to the ICESCR, but also to its own Basic Law and ethos of care through health, economic, legal, and social protection.

## Limitations

Firstly, the use of convenience sampling meant that the respondents may not have been fully representative of the African ASRs community in Hong Kong. However, the fairly large proportion of responses obtained which represented about one- third of the target population was reassuring. Secondly, this was a cross-sectional study which only permitted the determination of associations between factors and outcomes but not causation; results must be considered and interpreted accordingly. Lastly, while inpatient services were chosen as a proxy measure to represent acute or emergency healthcare utilization, we did not have data on the reasons for admission. Therefore, the appropriateness or inappropriateness of consultations cannot be properly assessed.

## Conclusion

Our study has shown that although a range of factors contributes directly and indirectly to ill health status of African ASRs in Hong Kong, there is an overwhelming need for better access to health for ASRs in Hong Kong. Furthermore that this can be rationalized on humanitarian grounds, through Hong Kong’s own commitment to their care-based healthcare, and on the basis of their local and international obligations. Despite this, the greater overarching forces that shape the lives and wellbeing of ASRs within Hong Kong’s community, that is government policy, cannot be underestimated. Of critical importance is a more transparent and accountable measures to improve the efficiency of screening of ASR claims, will minimise entrapment of ASRs in Hong Kong.

## Abbreviations

95 %CI: 95 % Confidence Interval
ASR: Asylum seekers and refugees
CSDH: Conceptual framework on the Social Determinants of Health
EDS: Everyday Discrimination Scale
ICESCR: International Covenant on Economic, Social and Cultural Rights
OR: Odds ratio
PHQ2: Personal Health Questionnaire-2
UNCHR: United Nations High Commission for Refugees
USM: Unified Screening Mechanism
WHO: World Health Organization
